# Locating carbon neutral mobility hubs using artificial intelligence techniques

**DOI:** 10.1038/s41598-024-62701-z

**Published:** 2024-05-29

**Authors:** Madiha Bencekri, Sion Kim, Yee Van Fan, Seungjae Lee

**Affiliations:** 1 Department of Transportation Engineering/Department of Smart Cities, University of Seoul, Seoul, Republic of Korea; 2 Environmental Change Institute (ECI), University of Oxford, Oxford, OX1 3QY UK; 3 Sustainable Process Integration Laboratory, NETME Centre, Faculty of Mechanical Engineering, Brno University of Technology, Brno, Czech Republic; 4 Department of Transportation Engineering, University of Seoul, Seoul, Republic of Korea

**Keywords:** Mobility hub, Sustainable transportation, Hub location problem, Genetic algorithm, Ensemble methods, Environmental sciences, Sustainability

## Abstract

This research proposes a novel, three-tier AI-based scheme for the allocation of carbon–neutral mobility hubs. Initially, it identified optimal sites using a genetic algorithm, which optimized travel times and achieved a high fitness value of 77,000,000. Second, it involved an Ensemble-based suitability analysis of the pinpointed locations, using factors such as land use mix, densities of population and employment, and proximities of parking, biking, and transit. Each factor is weighted by its carbon emissions contribution, then incorporated into a suitability analysis model, generating scores that guide the final selection of the most suitable mobility hub sites. The final step employs a traffic assignment model to evaluate these sites’ environmental and economic impacts. This includes measuring reductions in vehicle kilometers traveled and calculating other cost savings. Focusing on addressing sustainable development goals 11 and 9, this study leverages advanced techniques to enhance transportation planning policies. The Ensemble model demonstrated strong predictive accuracy, achieving an R-squared of 95% in training and 53% in testing. The identified hubs’ sites reduced daily vehicle travel by 771,074 km, leading to annual savings of 225.5 million USD. This comprehensive approach integrates carbon-focused analyses and post-assessment evaluations, thereby offering a comprehensive framework for sustainable mobility hub planning.

## Introduction

Sustainable mobility transcends the mere adoption of greener transportation modes. It represents a comprehensive shift in transportation planning, encompassing an environmentally, economically, and socially equitable movement of people and goods^1^. This shift has led to improved air quality, better health, and enhanced quality of life^2^. Transit-oriented development (TOD) is at the intersection of urban planning and sustainable mobility^3^. It seeks to create dense mixed-use communities centered around public transportation hubs^4^, aiming to create more environmentally friendly and socially vibrant urban spaces^5^. Following the principles of TOD, the ‘15-min city’ emerged as a new sustainable paradigm^6^, ensuring that all essential services are within a 15-min reach by foot or bike^7^. This model prioritizes pedestrian, cycling, and public transit infrastructure to drastically reduce car dependence and cut carbon emissions^8^, thereby fostering vibrant, healthy, and inclusive neighborhoods^9^. However, this concept faces significant challenges, notably displacing lower-income residents^9^ and the practicality of implementing this model across diverse urban landscapes. Cities with entrenched infrastructure or sprawling layouts may struggle to adapt, highlighting the necessity for context-sensitive strategies^10^.

Amid this landscape, mobility hubs present innovative solutions for advancing TOD, the 15-min city model, and sustainable mobility. Positioned strategically within urban spaces, these hubs can significantly diminish private vehicle dependence^11^, triggering a shift towards sustainable modes and fostering the development of a TOD and 15-min city model^12^. They are not just transit stops; they embody a holistic blend of transportation modes and offer other ancillary services, such as bike repairs, electric vehicle charging ports, retail outlets, and real-time travel information^13^. In San Francisco, mobility hubs successfully reduced the use of private vehicles. Vienna’s Aspern Seestadt hub, focusing on electric and shared mobility, promotes environmental sustainability. Toronto’s mobility green hubs incorporate green infrastructure, highlighting the dual benefits of improving the transport system while enhancing urban green spaces. Helsinki’s Herttoniemi mobility hub offers a blend of shared mobility and logistic services. These examples from around the globe underscore the critical role of mobility hubs in creating sustainable, efficient, and livable cities.

Although the ability of mobility hubs allows them to address diverse urban needs, the existing research has provided an incomplete view of their potential impact and optimal placement^14^. Michel et al.^14^ revealed that previous studies have mostly focused on travel time-based heuristic optimization, neglecting several other critical factors and methods. Meanwhile, Arnold et al.^11^ suggested using a more holistic methodology that includes additional built environment factors and a mix of optimization methods, including heuristic and GIS-based ones^11^. Specifically, the research gaps are as follows^11,14^:

1. Limited scope focus: overreliance on basic metrics such as population and employment densities without considering other factors, including transit access, parking, and carbon emissions.
2. Heuristic methods dependency: excessive use of heuristic approaches, which may overlook optimal solutions owing to inherent assumptions and complexities of real-world challenges.
3. Bias in decision-making: dependence on MCDM methods, which are prone to bias and subjectivity.
4. Travel time overemphasis: predominant focus on travel time in selecting mobility hub locations, neglecting essential environmental and operational factors.
5. Lack of post-assessment evaluation: absence of mobility hubs’ Impacts assessment and measurement.

This study advances SDGs 11 (sustainable cities and communities) and 9 (industry, innovation, and infrastructure) by focusing on mobility hubs as a means to foster TOD and the 15-min city model. The aim is to reduce car usage and emissions. This study demonstrates the role of mobility hubs in SDG 9 by employing innovative location-selection techniques and utilizing real-time traffic data to enhance efficiency. For SDG 11, the study underscores how strategically placed mobility hubs support sustainable urban areas where essential services are accessible within 15 min via eco-friendly transport. This promotes sustainable, efficient, and community-centric cities.

The significant role of artificial intelligence in advancing urban and mobility planning is widely acknowledged. Using AI in city planning helps enhance efficiency and sustainability, which can lead to smarter decisions and better services for everyone^15^. This study enhances mobility hub location research by uniquely combining genetic algorithms (GAs) with Ensemble methods and prioritizing parking capacity for optimization. GAs systematically evaluate potential solutions and optimize travel times, whereas Ensemble methods improve the accuracy of evaluations regarding carbon emissions. This innovative methodology addresses critical research gaps by incorporating often-overlooked elements, such as parking capacity, which significantly improves the accuracy and objectivity of the findings. This approach not only elevates the selection process but also establishes a new standard for rigor in the selection of mobility hubs. The paper is structured as follows. Section “Methodology” describes the methodology, Sect. “Results and discussion” discusses the findings and policy implications, and Sect. “Conclusion” concludes the study.

## Methodology

**Figure 1:**
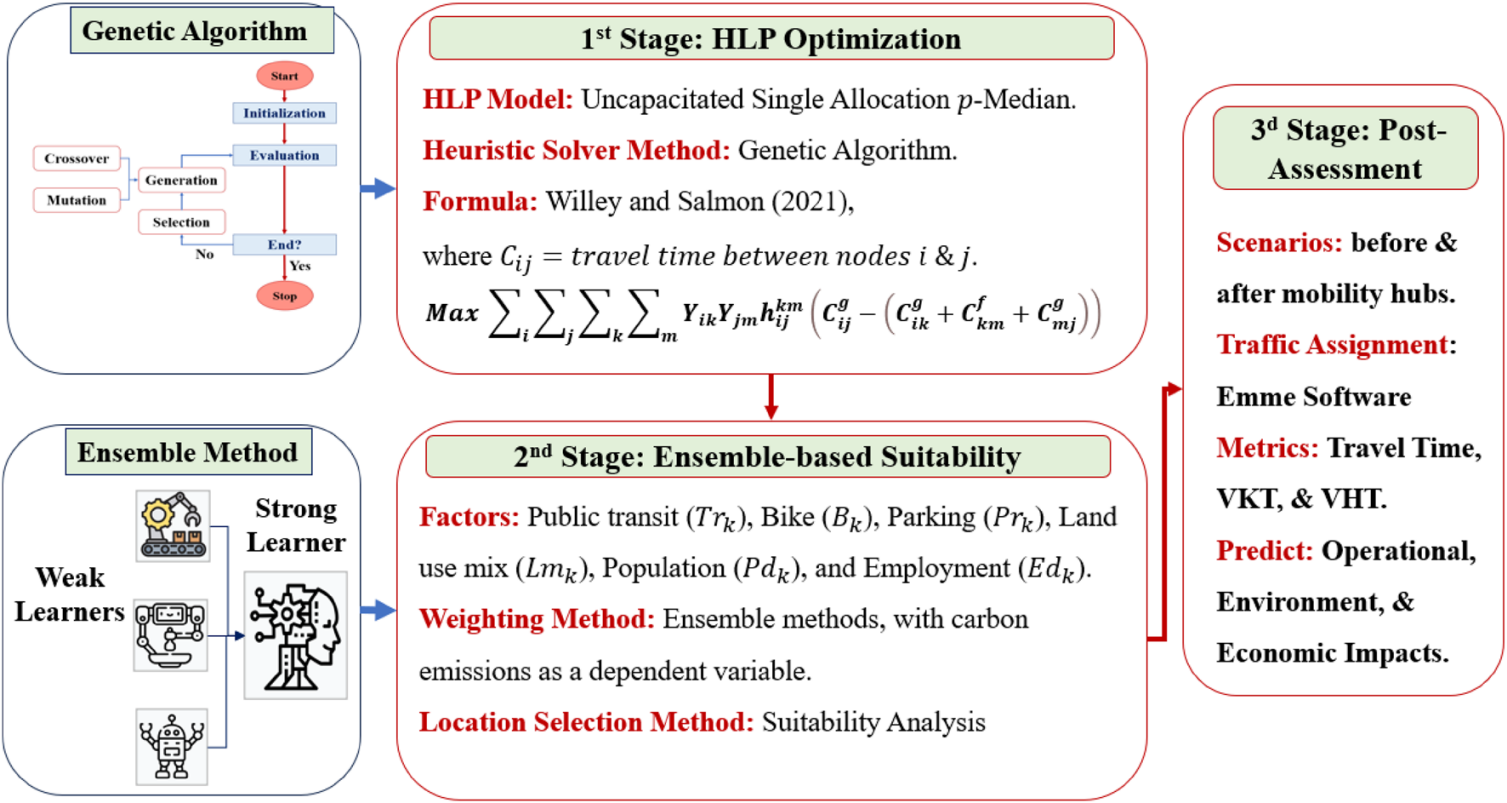
Proposed methodology for mobility hub location selection.

The proposed methodology employed a hierarchical three-stage approach. The first stage involves travel time-based optimization using GAs. The second step consists of selecting key multidimensional parameters, measuring their relative weights with respect to carbon emissions using Ensemble methods, and conducting the GIS suitability analysis (Fig. 1). The final stage focuses on implementing a quantitative post-assessment that uses traffic assignment to gauge the benefits of the selected locations in terms of travel time savings and reductions in carbon emissions.

### First-stage: GA optimization

To overcome the complexity of the hub location problem (HLP), this study employs GAs because of their proven capability to efficiently search for extensive solution spaces. GAs replicate natural selection processes using selection, mutation, and crossover techniques to evolve sets of solutions. These techniques iteratively enhance solutions based on their effectiveness in minimizing the costs and time associated with hub placement. The research implements GAs within an uncapacitated single-allocation p-median framework, aiming to select an optimal set of hubs from a pool of candidates and assign non-hub nodes to these hubs, thereby maximizing network efficiency. Starting with a diverse array of potential hub configurations, GAs systematically refine these through simulated evolution, favoring configurations that achieve lower transportation costs and higher efficiency.

### Second-stage: ensemble-based suitability analysis

This research distinguishes itself by deploying a sophisticated set of evaluative criteria that combine traditional indicators with the pioneering element of ‘parking proximity.’ Suitability analysis rigorously evaluates potential sites against these criteria, which are weighted according to their contribution to carbon emissions.

Advancing beyond the contributions of Aydin et al.^16^, this research adopts Ensemble methods such as Random Forest Regressor, XGBoost, and Gradient Boosting to weight factors based on their impact on carbon emissions, the dependent variable. This approach was selected for its effectiveness in handling multifaceted datasets and for minimizing bias. The weights of the factors are calculated using both the direct importance and permutation importance when direct measures are unavailable. Insights from various models were integrated to calculate consolidated mean importance scores, which were then standardized using z-scores. This approach enhances the interpretability of the findings and contributes to credible predictive analysis. The ensuing analysis provides a suitability analysis to strategically select locations that align with the environmental objective of reducing carbon footprints.

This study starts by transforming raster data, such as land use, to a vector format, and then calculates the Emsemble-based weights for factors, enabling district-level aggregation and normalization. It utilizes the ‘Business Analysis’ feature of ArcGIS Online owing to its accuracy and flexibility. A key innovation of this research is the inclusion of parking proximity and the application of Ensemble methods to weigh various factors with respect to their individual contributions to carbon emissions. This reduces bias in the selection process while addressing environmental concerns.

### Quantitative post-assessment

This study conducted a thorough assessment of the impact of mobility hubs on travel metrics, such as travel time, vehicle kilometers traveled (VKT), and vehicle-hour traveled (VHT). It leverages traffic assignment modeling to evaluate the chosen sites both before and after the implementation of these hubs. Employing the Emme software, this study highlights significant economic and environmental advantages. This methodological approach not only facilitates the validation of site selection for mobility hubs against pragmatic goals, but also demonstrates the hubs’ contribution to altering modal shares and reducing carbon emissions. The results of this analysis indicate notable economic benefits, including reductions in operational costs, travel time, traffic accidents, air pollution, and energy consumption. These benefits were quantified following the guidelines of the Korean preliminary feasibility study (2021 edition), which offers a comprehensive framework for evaluating the positive impacts of mobility hubs.

## Results and discussion

This section reveals the effectiveness of the chosen approach in enhancing the sustainability and efficiency of mobility hubs by examining their potential impact. This section then explores the study’s major contributions and policy implications.

### First stage of hubs’ locations selection

This study optimized the selection of mobility hub locations using a genetic algorithm (GA). The GA process begins by initializing a population and selecting individuals based on their fitness. It then applies crossover mutations to iteratively evolve solutions, thereby emphasizing the survival of the fittest. This method systematically improves the selection of locations by adjusting key parameters such as the number of generations and mutation rates, thereby enhancing the quality of solutions^17^. Through the parallel exploration of solutions, the GA significantly increases the likelihood of identifying optimal hub sites owing to its efficient site optimization based on evolving fitness values.

The application of the GA in this study focused on optimizing traffic flow by minimizing travel times, successfully achieving a significant fitness value of 7.7e7. The GA-selected sites consistently favored areas with high traffic, as illustrated in Fig. 5. For instance, the selected site in Seoul’s central region is located in a densely populated district that is known for its commercial and tourist prominence. In contrast, the site in the east-north region features several transit junctions and routes. Meanwhile, selected sites in the west–north, west–south, and east–south zones are districts with high population density, concentrated academic institutions, and a well-connected commuting network within the Gyeonggi province.

### Second stage of hub location selection

This study transcends the constraints of traditional MCDM tools for location selection by integrating Ensemble methods. These methods derive importance scores for various factors with carbon emissions as the dependent variable, thereby serving as weights for these factors. This approach not only enhances accuracy but also promotes sustainability and efficiency in the decision-making process.

#### Initial analysis

**Figure 2:**
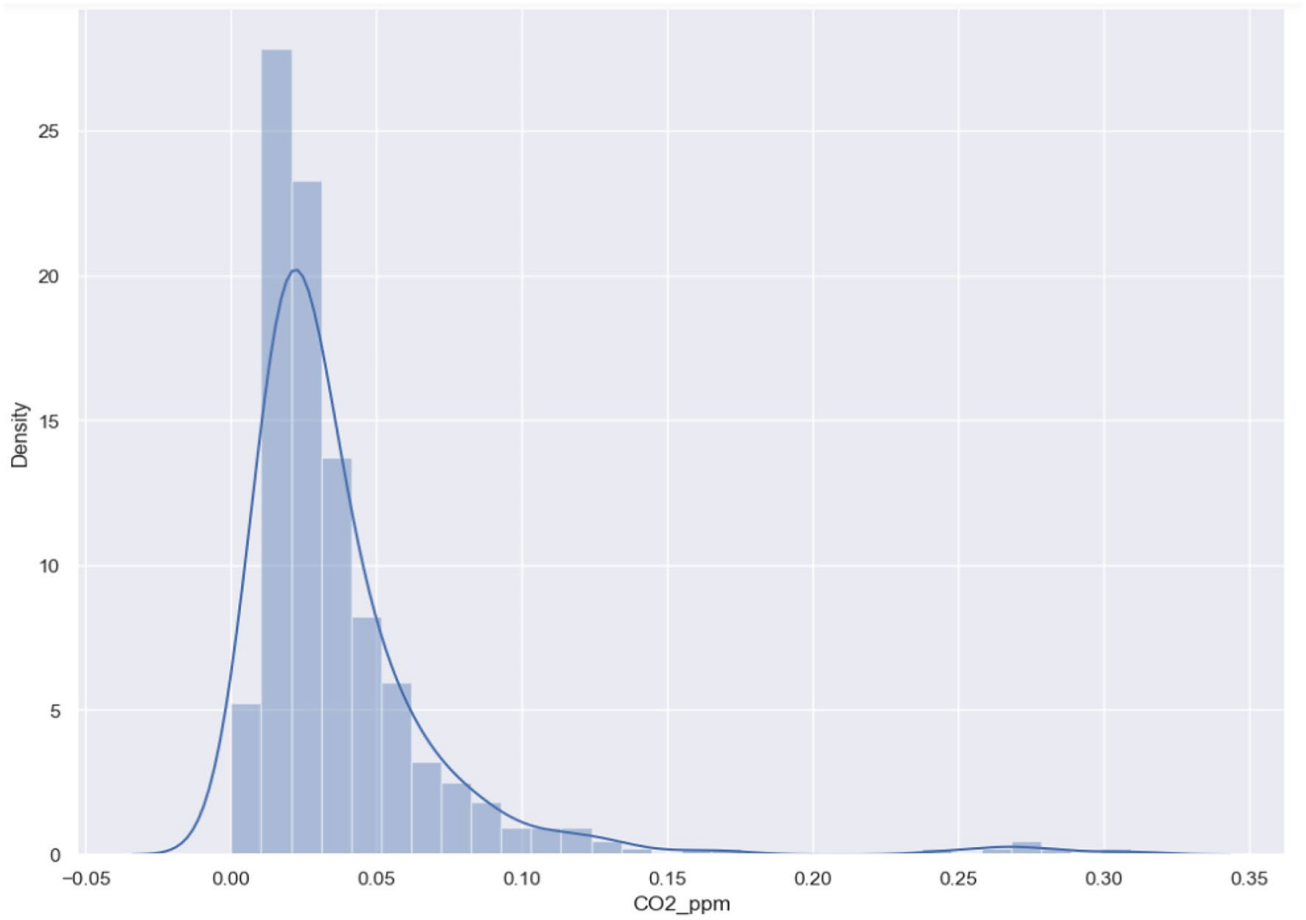
Carbon emission distribution.

**Figure 3:**
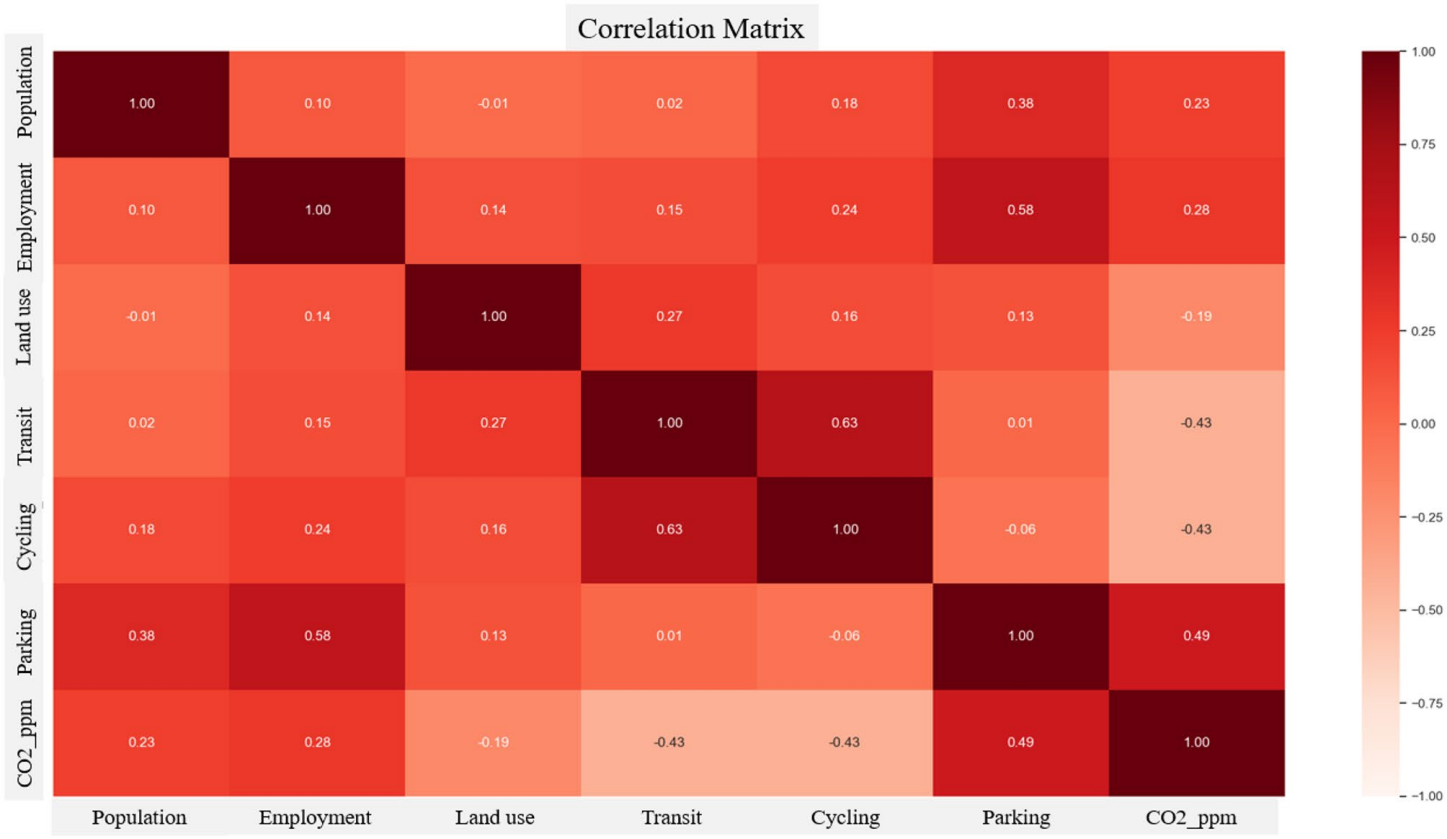
Correlation matrix.

The carbon emissions factor demonstrated a lognormal distribution, with most values being centralized around the mean (Fig. 2). These values predominantly ranged from 10 to 30 carbon units (CO2ppm). According to Fig. 3, the analysis indicates that the carbon emissions factor has a positive correlation of 0.49 with parking accessibility. Conversely, it shows a negative correlation of − 0.43 with public transit and cycling accessibility.

Beyond traditional methods such as London’s congestion pricing^18^, Bogotá’s TransMilenio bus lanes^19^, Copenhagen’s bike lane expansion^20^, and Prague’s dynamic parking pricing^21^, truly effective green transportation initiatives require a shift in focus. Central to this approach are the concepts of the 15-min city^22^ and TOD-oriented neighborhoods^23^, alongside the development of efficient and eco-friendly mobility hubs^24^. These strategies collectively underpin sustainable transportation systems that aim to mitigate environmental challenges while promoting healthier and more resilient communities. For these mobility hubs to effectively fulfill their roles, it is crucial to adopt a holistic strategy that not only enhances existing mobility infrastructure and services but also incorporates flexibility to respond to changing urban needs. This foundation supports the creation of hubs that are not only environmentally sustainable, but also dynamically responsive to the evolving landscape of urban mobility.

#### Ensemble-based weights calculation of factors

The study employed Ensemble methods to address the complexities of high-dimensional and multicollinear data, focusing on identifying key variables for accurate predictions. The data were split into 80% for training and 20% for testing, evaluating various algorithms, including the Decision Tree Regressor, Random Forest, AdaBoost. Notably, the Random Forest, Gradient Boosting, Bagging, and XGBoost Regressors stood out, with XGBoost showing exceptional performance, achieving an R-squared of 0.99 for training and 0.53 for testing, along with low error rates. Conversely, the Decision Tree and KNeighborsRegressor experienced overfitting and the MLP Regressor underperformed. The research then leveraged the top-performing models for GIS suitability analysis, demonstrating how Ensemble methods can enhance urban planning decision-making through comprehensive data analysis.

Although a 53% R-squared score might initially seem modest, it holds considerable significance in the context of urban transportation planning, where factors such as human behavior and urban development introduce substantial complexity. This score indicates that the models successfully accounted for over half of the variability in the data, providing a robust basis for making well-informed decisions in urban transportation planning.

**Figure 4:**
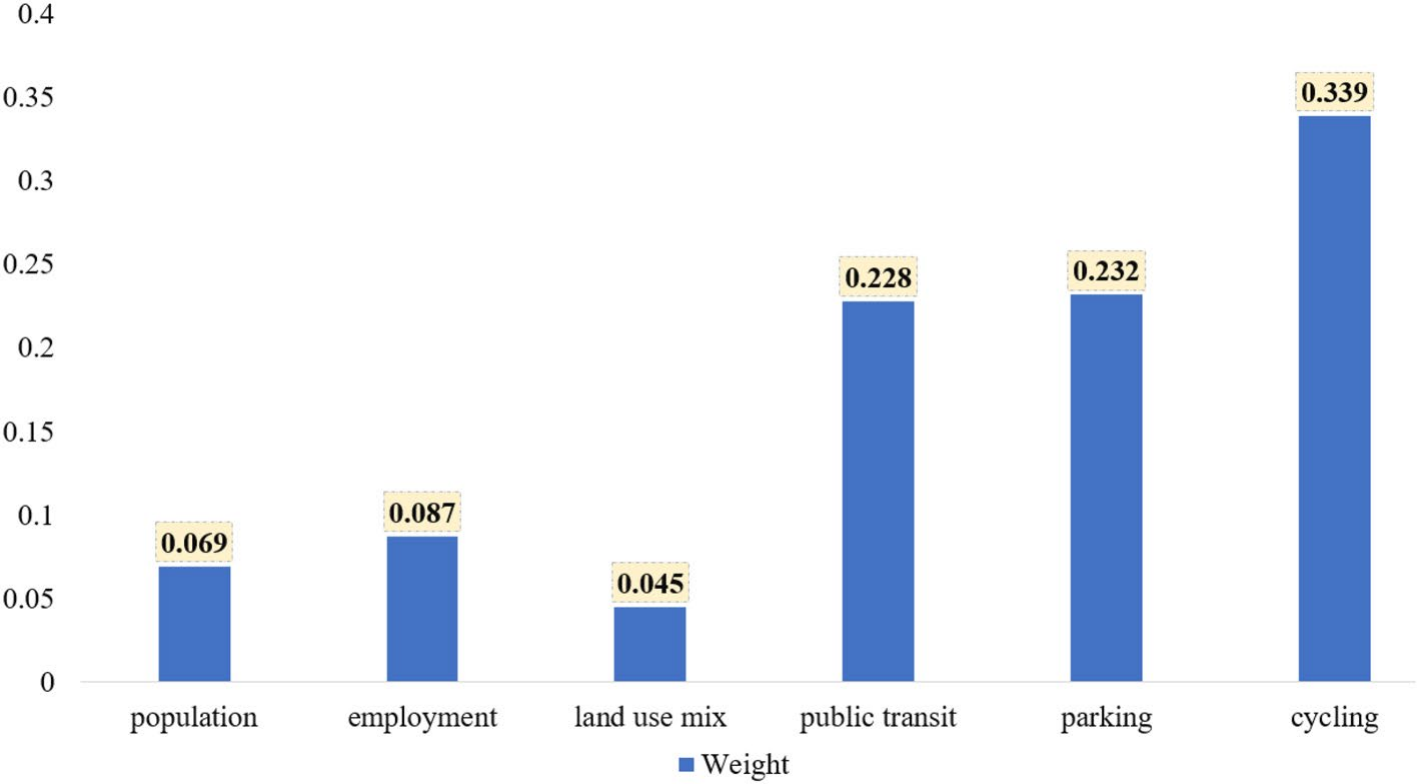
Factors’ importance scores (weights).

As illustrated in Fig. 4, the importance scores of the factors extracted from the best-performing Ensemble algorithms offer valuable insights into their contributions to carbon emissions. Cycling has the highest importance score of 0.3, indicating that promoting cycling can significantly reduce carbon emissions. Parking followed closely with a score of 0.232, emphasizing the importance of regulating parking spaces to reduce car dependency. Public transit has an importance score of 0.228, highlighting the role of well-connected and efficient public transit in reducing car usage, and consequently, carbon emissions. The land use mix and densities of population and employment have comparatively lower importance scores of 0.045, 0.069, and 0.087, respectively. Employment density plays a slightly stronger role, suggesting that job hubs influence mobility choices and, thereby, transportation emissions.

#### Suitability analysis

**Figure 5:**
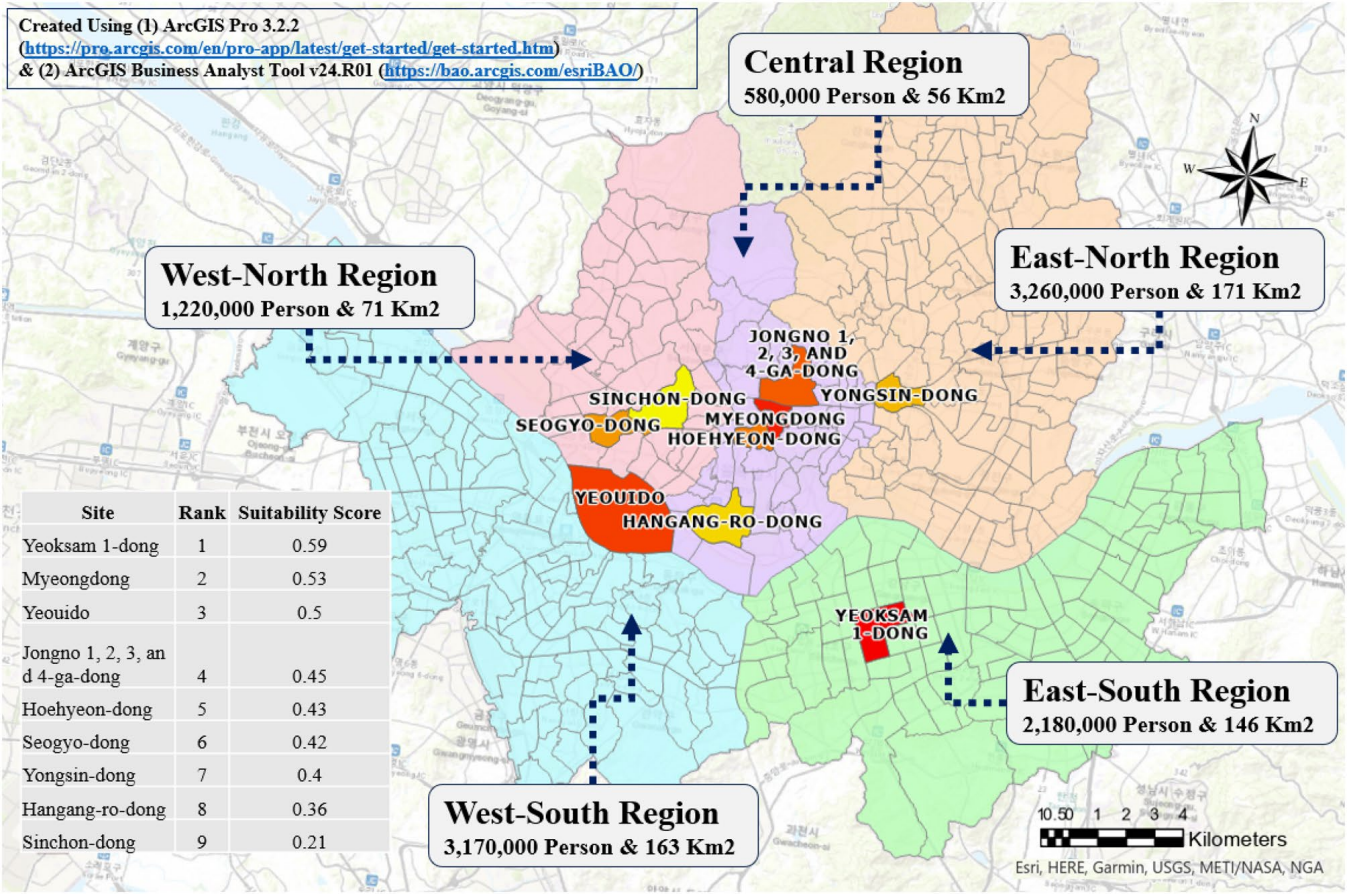
GA selected sites along with suitability rankings and scores.

In the selection phase, the previously calculated weight values are crucial for conducting suitability analysis and identifying the most sustainable hub locations. According to Fig. 5, the five districts with the highest suitability scores are (1) Yeoksam 1-dong, (2) Myeongdong, (3) Yeouido, (4) Jongno 1, 2, 3, and 4-ga-dong, and (5) Hoehyeon-dong. However, given that three of the top five selected sites were within the central region, this study further refined the selection by comparing suitability scores across different regions. This regional comparison led to the final selection of hub sites (Fig. 5), which included Yeoksam 1-dong in the east-south, Myeongdong in the center, Yeouido in the west-south, Seogyodong in the west-north, and Yongsindong in the east-north.

Yeoksam 1-dong, distinguished by its high concentration of job hubs and a comprehensive public transit network, presents a compelling case for the establishment of a mobility hub. Myeongdong, serving as both a commercial nucleus and cultural center for locals and tourists, necessitates advanced mobility solutions to accommodate its bustling activity and economic significance. Yeouido, the financial hub of South Korea, not only hosts paramount financial institutions but also features many leisure attractions and a well-connected transportation network. In Seogyo-dong, a district celebrated for its IT industry leadership, the introduction of mobility hubs can further foster innovation. Meanwhile, Yongsin-dong, a densely populated area with several educational establishments and a mix of residential and commercial spaces, exhibits diverse and high travel demands. The strategic integration of mobility hubs into these districts promises to enhance accessibility and support the distinct economic, cultural, and educational fabric of each area, advocating a nuanced approach to urban and mobility planning.

### Quantitative post-assessment

Sensitivity analysis was conducted to determine whether implementing mobility hubs in selected locations could effectively decrease congestion and carbon emissions. It was assumed that implementing a mobility hub would reduce travel time by 10%, followed by traffic assignment analysis using Emme software. The results indicated a significant shift in traffic flow: the use of private cars decreased on almost every road, favoring an increase in public transit modes, including bus, subway, or combined bus-subway trips (Table 1). Specifically, private vehicles and taxi trips showed a daily decrease of 37.75% and 14.22%, respectively, whereas the total number of buses, subways, and their combined trips increased by approximately 50% (Table 1).

**Table 1:** Modal shift before and after mobility hubs per day (percentages are calculated magnitude-wise).

Mode	Number of trips—before	Number of trips after	Difference	Difference in %
Automobile	21,196,831	21,146,175	− 50,656	− 37.75
Taxi	3,171,534	3,155,092	− 16,442	− 12.25
Bus	6,995,355	7,014,433	19,078	14.22
Subway	5,916,110	5,949,049	32,939	24.55
Combined subway & bus trips	2,833,118	2,848,199	15,081	11.24
Total modal shift			67,098	

These findings, displayed in Table 1, demonstrate that mobility hubs have effectively reduced reliance on personal cars and taxis, while promoting the usage of public transit modes. The combined subway and bus modes also saw an increase, indicating the hub’s effectiveness in facilitating multimodal journeys. Therefore, mobility hubs have successfully influenced modal choices, steering users towards more sustainable and efficient modes of travel.

In this study, a traffic assignment model was applied to measure the changes in VKT and VHT before and after the introduction of mobility hubs. These metrics are crucial in calculating key economic advantages, including operational cost savings, travel time savings, safety-improving benefits, and reduction in air pollution. The results showed a reduction of 771,074 VKT per day, demonstrating that these hubs have effectively influenced modal choices by making public transport or other sustainable modes more appealing, as confirmed by the modal shift (Table 1). Similarly, there was a decrease of 37,056 VHT per day, suggesting an improvement in trip efficiency due to reduced congestion and more direct routes facilitated by the implemented hubs.

Table 2 presents the economic benefits accrued from the introduction of mobility hubs following the instructions of the Korean feasibility study guidelines. The results indicated annual operational cost savings of approximately 65.2 million USD, largely due to more streamlined operations, decreased maintenance, and efficient use of transportation resources as hubs integrate and connect various modes. Annual travel time savings were around 153.75 million USD, reflecting the hubs’ roles in minimizing delays and facilitating faster commutes. Additionally, hubs have fostered safer transportation, with annual savings of approximately 3.1 million USD, mostly due to improved road use, modal transfer, and reduced congestion. On the environmental front, the hubs contributed to a reduction in air pollution, with annual savings of over 3.8 million USD, mainly attributed to decreased private vehicle usage and shifts to public transit. Consequently, the establishment of these mobility hubs generated significant annual economic gains, totaling 225.9 million USD.

**Table 2:** Mobility HUBS’ implementation benefit (USD per year).

Savings	Operation cost saving	Travel time saving	Accident savings	Air pollution savings	Sum
Value (USD)	65,215,049.44	153,759,452.10	3,132,224.33	3,833,968.85	225,949,054.66
Percentage	28.86%	68.05%	1.39%	1.70%	100%

This study delved into the outcomes of implementing mobility hubs with a focus on the impact of the methodologies used for site determination. Employing GAs, the research optimized travel times, favoring high-traffic areas in Seoul. Recognizing the need to consider additional environmental aspects, the second stage utilized Ensemble methods to (1) incorporate several key factors and (2) account for carbon contributions to the decision-making process. This provided a comprehensive approach for site selection. These findings identified factors such as cycling and parking proximity as significant contributors to carbon emissions. Following the application of GA, the Ensemble-based suitability analysis further refined site selection. A quantitative post-assessment confirmed the positive impacts of mobility hubs, showing significant reductions in the modal share of private vehicles as well as in the VKT and VHT metrics. The research estimated the calculated economic benefits to be approximately 225.9 million USD annually. This study intends to bridge theoretical methodologies with practical urban benefits to guide future sustainable urban and transportation planning initiatives.

### Policy recommendations

Beyond traditional considerations of travel time in selecting mobility hub locations, this study employs an Ensemble-based suitability analysis that incorporates each factor’s contribution to carbon emissions. It integrates cycling, parking, and public transit proximity to determine optimal sites for mobility hubs. Additionally, a post-assessment quantitatively evaluates the environmental and economic benefits using traffic assignment modeling. Given the comprehensive findings, this study advocates for the following policies:

1. Considering various factors:
  Incorporating a range of factors beyond travel time for a more realistic approach, encompassing socioeconomic, land use, transportation infrastructure, and environmental criteria.
  Tailoring these considerations to the unique characteristics of each city, incorporating the key elements influencing travel patterns.
2. Leveraging AI and new technologies in decision making:
  Utilising AI techniques to optimize site selection and predict possible outcomes.
  Implementing IoT and big data abalytics to increase the accuracy of the optimization, and to continusouly monitor and refine mobility hubs functioning.
  Employing AI tools to visualize different scenarios and facilitate stakeholders discussion and participation.
3. Implementing ensemble-based suitability analysis:
  Utilizing ensemble methods to ensure non-biased decision-making.
  Applying these methods to achieve various goals, such as reducing carbon emissions, relieving congestion, and enhancing physical activity.
  Customizing the reflection of carbon emissions in factors’ weights for the identification of mobility hub sites.
  Implementing these carbon-based weights permits to quantify reductions in carbon emissions, supporting cities in achieving environmental goals like those outlined in the Paris agreement.
4. Selecting hubs sites: must-included characteristics:
  Considering cycling proximity in the selection of hub locations, as it significantly reduces carbon emissions.
  Establishing a cycling network connecting major residential and commercial areas to hubs, ensuring safe and direct routes for cyclists, integrated with features like bike parking, repair facilities, and bike-only lanes.
  Recognizing the significance of parking proximity in reducing carbon emissions, by integrating parking proximity in the site selection process and including parking spaces in mobility hubs to discourage car use.
  Implementing parking policies such as dynamic parking pricing and limited parking authorization to deter car usage and generate revenues.
  Integrating active mobility (cycling and pedestrian facilities) and public transit modes within mobility hubs to strengthen their attractiveness and usage convenience.
  Promoting policies such as improving modal integration and providing financial incentives to choose active and public transit modes.
5. Continuous monitoring and assessment:
  Gathering qualitative insights through user satisfaction surveys to evaluate travel experience at mobility hubs.
  Tracking quantitative measures such as changes in public transit ridership, reduction in private vehicle usage, air quality improvements, and public health metrics.
  Systematically incorporating user feedback and quantitative real-time data into mobility planning.
  Employing a dynamic approach to evaluation and adaptation, allowing mobility hubs to evolve in response to changing needs and conditions, and ensuring their long-term effectiveness.

By adopting these policy recommendations, cities can progress towards developing efficient, sustainable, and accessible mobility hubs, thereby significantly enhancing their sustainability and efficiency.

## Conclusion

Mobility hub location selection is pivotal for sustainable urban development. Although previous research has provided valuable insights, there has been a predominant focus on travel time, the use of heuristic methods, and prone to biased spatial decision-making. This study addressed these gaps by presenting a comprehensive three-tiered hierarchical approach that integrates a wide range of factors and combines the power of the heuristic GA and Ensemble methods to enhance decision-making by reducing bias and improving accuracy.

The initial phase utilized the GA, achieving a notable fitness value of 7.7e7, to optimize travel time in selecting potential hub sites. Subsequently, an ensemble-based analysis assigned weights to factors based on their contributions to carbon emissions impact and implemented GIS suitability analysis using these weights. The Ensemble methods achieved an R-squared score of 0.95 on training data and 0.53 on test data, which, despite appearing low, is significant in transportation planning contexts due to complex variables like human behavior and urban development. A post-assessment using traffic assignment analysis quantified the benefits of the selected sites, revealing substantial travel time savings, significant reductions in carbon emission, and other economic advantages. Assuming a 10% reduction in travel time following hub implementation, this study found a marked decrease in the use of private cars and taxis, alongside an increase in public transit and cycling usage. Additionally, the economic benefits were substantial, with a total savings estimated at 225.5 million USD annually. These findings highlight the effectiveness of mobility hubs in enhancing cities’ efficiency and sustainability.

This study’s framework focuses on carbon-centric solutions for sustainable mobility hubs, aligning with SDGs, TOD, and the ‘15-min city’ concept. It is distinguished by its methodological innovation in selecting sustainable sites for mobility hubs. While the findings are primarily based on data from Seoul, limiting broader generalization, the methodology itself is versatile and applicable to any urban context. However, the study’s quantitative focus might overlook essential qualitative insights such as hubs’ physical features and the cultural and historical contexts. Consequently, future research should expand to consider a wider range of urban, environmental, and social factors. This expansion includes involving the community in the final decision-making, employing more robust HLP solvers and predictive methods, and using multiple dependent variables to capture not only the impact on carbon emissions, but also safety, social equity, and other critical planning metrics.

### Supplementary Information

Supplementary Information.

## Data Availability

The datasets used and/or analyzed during the current study are available from the corresponding author on reasonable request.
