# Supplementary material for: Locating carbon neutral mobility hubs using artificial intelligence techniques
Source: Sci Rep. 2024 May 29;14:12328. doi: 10.1038/s41598-024-62701-z (PMC11137099; doi:10.1038/s41598-024-62701-z)
Supplement: Supplementary file 1 — Supplementary Information. [file 41598_2024_62701_MOESM1_ESM.docx]

Supplementary File

# **Title:** Optimizing Carbon-Focused Allocation of Mobility Hubs Using Genetic Algorithms and Ensemble-based Suitability Analysis

# Annex A: Literature Review:

This study addresses the complex challenge of selecting locations for mobility hubs, to enhance decarbonization, and thereby sustainability. Recognizing current gaps, this research focuses on defining key parameters that influence mobility hubs’ performance, considering not just traditional factors, but also emphasizing the reduction of urban carbon emissions and the proximity to parking spaces. By employing genetic algorithms and Ensemble-based GIS suitability analysis, the study offers a novel methodology capable of deeply analyzing these parameters, thereby contributing to more sustainable and informed mobility hub sites’ selection practices.

## Identification of Parameters

Travel time is predominantly recognized as the principal cost attribute in mobility hub location selection ^1^. Fan et al. (2018) ventured into integrated location optimization, considering factors such as network operating costs, transformation costs, and hub construction outlays ^2^. However, the absence of environmental considerations and the model’s exclusive applicability to highways further limit its comprehensiveness. Willey and Salmon (2021) embarked on delineating locations for vertiports, but their framework focused on total transportation costs and lacked environmental perspectives ^3^. This prevailing focus on the ‘travel time’ metric tends to eclipse the importance of other vital indicators, most notably those related to the environment such as carbon emissions. For instance, Arnold et al. (2023) highlighted a discernible literature lacuna concerning environmental indicators ^4^, calling for a more holistic approach that integrates carbon emissions and implements several built environment factors. The suggested additional factors refer to human activity, including parking lots, land use mix, and bikes and transit stations ^5^. Advocating for a holistic approach that includes a wide range of criteria such as carbon emissions reduction and parking infrastructure, this study advances the methodology for mobility hub location selection, aiming to contribute to more sustainable and livable cities.

Parking availability can significantly influence mobility hub usage. Navratilova et al. (2021) explored the application of a mobility hub with automatic parking. However, they focused on understanding how automatic parking within a mobility hub can enhance parking availability and reduce congestion in cities ^6^ . Kirschner and Lanzendorf (2019) reviewed existing policies and challenges related to parking management and emphasized the need for parking policies to be considered an integral part of sustainable transportation strategies ^7^. Implementing parking facilities in mobility hubs allows for the attraction of car users to public transit. However, it is essential to acknowledge the challenges related to high costs associated with the installation and maintenance of parking spaces, the risk of perpetuating car dependency, and potential social equity issues.

Proximity to public transit is a fundamental factor in determining mobility hubs’ sites. Transit stations provide a convenient and reliable way for people to get to and from the hub, and they can also help attract riders from surrounding areas. Storme et al. (2021) presented a comprehensive model for ranking candidate locations of transit hubs. Their model considered various aspects, including the overall efficiency of the transit network transfer intensity, proximity to major passenger generators/attractors, hub service coverage, compatibility with land use restrictions, and adaptability to future transit concepts ^8^. Nevertheless, integrating transit stations into existing urban settings involves navigating spatial constraints, and zoning regulations, and adapting to the established built environment, a process fraught with challenges including space allocation conflicts and the need to maintain the area’s characteristics. Successful integration demands strategic planning and proactive stakeholder engagement to ensure that these new infrastructures complement the urban fabric without displacing or disregarding the needs of existing communities.

Cycling can bridge the first and last-mile gaps, making the interplay between cycling services and public transit an important factor in urban mobility ^9^. Thus, integrating bike-sharing into mobility hubs can enhance the hubs’ accessibility, convenience, and sustainability ^10^. Pase et al. (2020) considered the integration of bike-sharing with public transit as a factor that can expand the potential of both modes of transportation ^9^. Addressing the maintenance requirements of bikes and docking stations is vital for the reliability and user satisfaction of bike-sharing systems, necessitating regular upkeep to ensure both safety and functionality. Moreover, the logistical challenge of redistributing bikes to meet demand across different city areas is crucial for maintaining service efficiency and accessibility. Seasonal variations in usage also present significant challenges, as demand can fluctuate widely, affecting operational planning and resource allocation. Consequently, these systems must be agile and responsive, employing data-driven approaches to manage the complexities of maintenance, redistribution, and seasonal demand effectively.

Moreover, land-use planning, transit-oriented development (TOD), and mobility hubs are essential in crafting sustainable urban environments. Land use organizes the spatial distribution of activities across the urban landscape, aiming for an a well-organized city layout ^11^. TOD takes this a step further by strategically locating the high-density, mixed-use areas around public transit stations ^12^. This approach not only reduces reliance on automobiles but also promotes healthier, more active lifestyles through increased walkability, bikeability, and public transit usage ^12^. Finally, mobility hubs complement these efforts by consolidating various modes of transport to improve connectivity and accessibility ^13^. Together, these three strategies promote a symbiotic relationship: land use and TOD increase the efficiency and appeal of public transit, while mobility hubs improve access and convenience, creating vibrant, sustainable communities. By fostering such integration, cities can achieve a balanced development that supports environmental sustainability, social equity, and economic vitality.

## Mobility Hubs Allocation Methods

The fusion of Genetic Algorithms (GAs) with Geographic Information Systems (GIS) and Multi-Criteria Decision-Making (MCDM) methods offers a robust framework for solving the Hub Location Problem (HLP). This problem, aimed at finding the best hub locations to minimize costs and enhance service quality and coverage, is recognized for its computational complexity due to its NP-hard status ^14^. The intricate challenge of determining optimal placements for mobility hubs is accentuated by the need to consider node placement and the overall network design intricacies ^15^.

Historical insights into HLP, including O’Kelly’s distinction between single and multiple allocation models ^16^, and Campbell’s classification into four HLP types, underscore the critical role of strategic hub placement in streamlining spatial flows and reducing costs ^17^. Notably, The uncapacitated single p-median model finds the best location for p hubs within a network to minimize overall travel times between the facility and users, without considering the facility’s capacity constraints ^17^. Urban transportation needs to determine the most effective site for a mobility hub, thereby optimizing access for users and streamlining the transportation network’s efficiency. To navigate the computational demands of HLP, especially in large-scale applications, Genetic Algorithms have emerged as a viable heuristic approach ^18^. By mimicking evolutionary processes, such as selection, crossover, and mutation, GAs adeptly explore potential solutions, enhancing the prospects of identifying optimal hub locations. This method, underpinned by Agent-Based Modeling (ABM), enables a thorough exploration of outcomes.

GIS technology complements this approach by providing advanced spatial analysis tools crucial for evaluating hub site feasibility, focusing on factors like population density and infrastructure availability ^19^. Combined with MCDM methods, which facilitate a structured comparison of location alternatives through approaches like the Analytic Hierarchy Process (AHP) and the Technique for Order Preference by Similarity to Ideal Solution (TOPSIS), this integrated approach supports a comprehensive and sustainable decision-making process ^20^. However, the MCDM methods rely on the input of decision-makers to define the criteria, assign weights, and evaluate the alternatives, introducing bias and subjectivity and potentially leading to suboptimal or biased location selections ^21^.

Additionally, incorporating Linear Programming (LP) with GIS illustrates the method’s versatility in optimizing hub locations against diverse criteria. Arnold et al. (2022) stated that Spatial analysis plays a crucial role in deciding where to put mobility hubs, proposing a conceptual framework that contains four key elements: purpose, process, place, and performance ^4^. So et al. (2023) used spatial data to emphasize the strategic importance of incorporating sustainability criteria into the selection process of mobility hubs ^19^. The integration of GIS with Linear Programming (LP) in addressing the logistics hub location problem, as demonstrated by Shahparvari et al. (2020), further illustrates the versatility of GIS in solving complex hub location challenges. This study first identified potential areas with GIS and MCDM tools, then selected the best site with a K-means heuristic and PROMETHEE methods ^22^. Peker et al. (2016) emphasizes the significance of considering spatial proximity, dispersion, and node importance, reinforcing the utility of GIS in facilitating detailed spatial analyses to identify optimal hub locations ^23^.

This integrated approach, marrying the heuristic power of GAs with GIS’s spatial analysis, represents a significant leap forward in transportation planning. It not only aids in finding near-optimal solutions to the HLP but also ensures these solutions are practically and spatially informed, promoting sustainable and efficient infrastructure development.

## Research Gaps and Contributions

Several studies (Table 1) have focused on travel time as the primary cost attribute in the selection of mobility hubs’ locations, often overlooking other crucial factors, such as carbon emissions. Arnold et al. (2023) called for a more comprehensive approach, a sentiment echoed by Michel et al. (2022), who advocated for the inclusion of a broader range of multidimensional factors in the location selection process ^4,5^. The solving techniques were based either on heuristics methods or by MCDM-based GIS analysis. Deluka-Tibljaš et al. (2013) highlighted the subjectivity of MCDM methods, suggesting the need for more objective and accurate tools ^24^. This sentiment was echoed by Anderluh et al. (2020), who pointed out the bias inherent in AHP, which is a commonly used MCDM method in mobility hub location selection ^25^. While Shahparvari et al. (2020) proposed integrating both GIS analysis with Linear programming in addressing hub location problems ^22^.

Table 1 provides a comprehensive review of various studies related to mobility hub location, highlighting each study’s unique contributions and the limitations they identified. The table culminates with the contributions of the current study, emphasizing its novel approach, the integration of multiple factors, and its efforts to address previously identified gaps in the literature. The following research gaps are identified:

- Neglect of a holistic view of built environment factors, with a narrow focus on typical metrics like population and employment densities, while overlooking transit, parking proximity, and environmental aspects such as carbon emissions ^5^.
- Excessive dependence on heuristic methods, which might not capture the optimal solutions due to their intrinsic assumptions and the intricacies of real-world challenges ^3,26^.
- Apparent biases and subjectivity in decision-making tools, especially when using MCDM-based spatial optimization methods and techniques like the analytic hierarchy process ^27^.
- Complete omission of parking capacity and its proximity as influential factors in the mobility hub location selection process, even though they play a pivotal role in determining hub efficacy ^6^.
- Overemphasis on travel time as the key determinant in mobility hub location choices, often at the expense of other vital environmental and operational considerations ^4^.
- Lack of comprehensive post-selection assessments for mobility hub locations, resulting in missed opportunities to gauge their real-world benefits and implications ^8^.

Table 1: Literature Contributions and Gaps

| **Study** | **Identified Gaps** |
| --- | --- |
| (Deluka-Tibljaš et al., 2013) | Pointing out the limitations of the MCDM methods and suggesting the need for more objective and accurate tools. |
| (Ratti and El-Geneidy, 2017) | Focusing on travel time as the only element of the used cost, while overlooking environmental, built environment, and socioeconomic factors.  Ignoring the impact of parking proximity on mobility hubs’ efficiency. |
| (Fan et al., 2018) | Only considering the network operating cost, network transformation cost, and hub construction cost, overlooking environmental and other costs.  Relying primarily on a heuristic method to provide the optimal solution. |
| (Anderluh et al., 2020) | Depending on the AHP method for hub location selection, bypassing its subjectivity and bias in criteria weighting |
| (Tippabharlta, 2020) | Anchoring on travel time as the main cost attribute, omitting environment elements. |
| (Kaveh et al., 2021) | Selecting the best locations based on the following parameters: the hub's capacity and construction costs, and transportation time and demand.  Neglecting the impact of environmental factors. |
| (Willey and Salmon, 2021) | The study identified desirable locations for vertiports while considering vehicle limitations desired operational strategies, and the possibility of passenger trips involving more than two vertiport trips.  The results are only based on meta-heuristics methods, which might not always provide the optimal solution.  The study did not consider any environmental parameter or impact. |
| (Arnold et al., 2023) | The study conducted a review of mobility hub literature.  The study highlighted the neglect of energy consumption and carbon emissions in the existing literature.  The study pointed out the need for a more comprehensive approach. |
| (Michel et al., 2022) | The study conducted a review of the mobility hub location optimization.  The study highlighted the need for a broader range of multidimensional factors in the location selection process for mobility hubs.  The factors include public transit facilities, micromobility and shared mobility modes and their parking, charging facilities for electric vehicles and e-scooters, etc. |
| (Ratli et al., 2022) | The study proposed a new heuristic method to solve the hub location routing problem.  The method outperformed existing algorithms and obtained new best-known solutions in less time.  The method is focused on comparing algorithms. |
| (Shin et al., 2022) | The study proposed a novel hub location problem (HLP) to plan a Sky port network for air taxi transportation systems, considering midair congestion.  The solution is solely based on the metaheuristic algorithm, which does not guarantee it is the optimal solution, as it is based on many assumptions.  The study did not investigate the environmental impact. |
| This Study | The study introduces a 2-stage optimization process with multiple multidimensional factors.  The study is the first to consider parking capacity as a factor player in the location selection.  The study is the first to integrate, at once, all potential built factors in the location optimization.  The study reflected the environmental aspects in the weights of the selected indicators.  The study used the Ensemble methods to measure the weights of the parameters, avoiding the subjectivity and bias of the MCDM methods and reflecting sustainability.  The study strengthened the selection by combining powerful methods: a genetic algorithm to solve the HLP and an Ensemble-based suitability analysis to compensate for the GA problems. |

# Annex B: Methodology & Results

Data for the analysis, sourced primarily from the Seoul metropolitan government’s open data portal, include road networks, station locations, land use, and socioeconomic information (Table 2).

Table 2: Input Data

| Data Type | Scale | Unit | Source |
| --- | --- | --- | --- |
| Road and Rail Network | All Seoul | km | Korea Transport Database (KTDB) |
| Bike and Transit Coverage | All Seoul | Location | Seoul Open Database |
| Land use (3rd level) | By land use type | Type or use |  |
| Population | By district | Person |  |
| Employment | By district | Job offers’ number |  |
| Parking | By district | Capacity |  |
| Carbon emissions | By big district | Parts per million |  |

## Hub Location Problem

The Hub Location Problem (HLP) aims to identify the best locations for hubs, targeting cost reduction, maximizing coverage, or enhancing service quality ^14^. The complexity of HLP is underscored by its status as a [nondeterministic polynomial-time](https://en.wikipedia.org/wiki/NP_(complexity)) hard (NP-hard) problem, indicating its intense computational demand. As the size of the network increases, the difficulty of resolving the problem increases. This complexity is further magnified when considering node placement intertwined with broader network design ^15^. Central to HLP are three components: hubs (target nodes for placement), the OD matrix (depicting the flow dynamics of goods or data), and a connectivity framework linking hubs to both hub and non-hub nodes ^14^. O’Kelly (1986) differentiated between single and multiple allocation scenarios. The former ensures that each node (origin or destination) connects to just one hub, whereas the latter permits flow through multiple hubs, introducing greater complexity ^28^. Building on this, Campbell (1994) offered an integer-focused approach to HLP, categorizing it into four specific types: (1) basic HLP, (2) $p$-hub median problem, (3) $p$-hub center problem, and (4) hub covering problem ^29^.

HLP is crucial in mobility hub site selection by aiming to optimize spatial flow routes and minimize costs. Gelareh and Nickel (2011) applied HLP to liner shipping yielding high-quality solutions ^30^, while Ishfaq and Sox (2011) examined intermodal hub network design and its impact on reducing transportation costs ^31^. Notably, when considering the intricate task of selecting the best locations for mobility hubs, where cost-effectiveness is paramount, uncapacitated single $p$-median allocation emerges as a robust approach. This model addresses both the intricacies of node placement and the overarching goals of HLP, making it a prime contender for effectively resolving the challenges presented by mobility-hub location decisions.

## Genetic Algorithm (GA) Introduction

HLP is a complex NP-hard optimization tool used to determine the optimal locations for hubs by minimizing transportation costs and time. Given the complexity, especially in larger real-world scenarios, exact algorithms might be computationally expensive or even infeasible owing to the large solution space and nonlinear nature of the problem. Consequently, heuristic algorithms have been commonly used to find good but not necessarily optimal solutions ^32^. They become particularly valuable when deriving the absolute best solution, either from computationally overwhelming or overly prolonged optimization challenges ^33^. They use rules of thumb, intuition, and experience to guide the search for solutions. Heuristic methods have been used extensively in diverse optimization problems. For instance, determining the optimal locations for alternative fuel stations with limited capacity ^34^, finding the most eco-friendly shortest path ^35^, and locating electric bus depots enhances efficiency ^36^ .

GA is a heuristic method that has emerged as potent tools capable of efficiently navigating the vast solution landscape of HLP. They applied the dynamics of evolution and natural selection to solve complex problems, which is beneficial for addressing NP-hard problems such as location selection challenges, as they focus on finding near-optimal solutions rather than solely aiming for early convergence and pinpointing optimal locations ^11^. GA represents a specialized branch of computational techniques within the expansive family of Agent-Based Modeling (ABM), a paradigm that models individual agents and their interactions within a system to study complex behaviors and emergent properties ^37^. ABM simulates the decisions and interactions of autonomous agents, making it invaluable for understanding dynamics in systems ranging from ecosystems to markets ^38^. Each ‘agent’ in GA embodies a potential solution, and through evolutionary processes akin to natural selection, these agents compete and collaborate, thus mirroring the interactive essence of ABM ^39^. The distinctiveness of GA lies in its methodological underpinnings, by encoding solutions as chromosomes, it allows for a rich exploration of the solution space through genetic operators. This evolutionary cycle, where agents undergo selection based on fitness, crossover to combine desirable traits, and mutation to introduce variation, exemplifies an adaptive dynamic system reflective of ABM’s core principles. And by mimicking the natural selection process, GA creates a population of potential solutions that undergo selection, crossover, and mutation to generate new generations. This allows for a more comprehensive exploration of the solution space, thereby increasing the likelihoods of finding the best optimal and near optimal solution solutions.

## First-Stage: GA Optimization

The uncapacitated single allocation $p$-median HLP refers to the selection of $p$ number of hubs within a λ set of possible candidates while allocating the non-hub nodes, in an ϒ set of possibilities, to one hub. In this case, it is assumed that the link between two hubs is less costly than the one involving non-hub nodes, all possible flow must pass by a hub, and all involved nodes are connected.

The HLP is formulated as a graph problem $G=(N, L)$ with node set $N=\{1, 2, \ldots.,n\}$, and $L$ reflects the link between pair of nodes $(i, j)$, and which is often represented by the arc length $l_{ij}$, and the arc flow $h_{ij}$ (Campbell and O’Kelly, 2012). When a transmission pair involves non-hub nodes, the service cost is $C_{ij}\geq0$. If both nodes are hubs, they provide the service at a potentially reduced cost, implying that investing in hubs can lead to cost savings. Applying a discount factor ($0<\alpha<1$), the reduced cost is ${\alpha C}_{ij}$. Every transport service consists of various transmission units, with the total cost being their combined sum. In the case of multiple allocation HLP, the cost includes three parts: cost of transferring goods from the origin node to this hub, transferring cost between two hub nodes, and transferring cost from the hub to the destination node. The decision variable $Y_{ik}$ of a node $i$ to hubs, where $Y_{ik}=1$  if a node $i$ is allocated to the hub $k$. The variable $X_{j}$ is set to 1 if there's a hub facility at node j, and 0 if not.

The cost between a pair of nodes $(i, j)$ via hubs $(k,m)$, is expressed as follows:

$$\begin{aligned} C_{ij}^{km}= C_{ik}+ {\alpha C}_{km}+ C_{mj} \#\left( Eq1 \right) \end{aligned}$$

Therefore, the mathematical structure of the uncapacitated single allocation $p$-median HLP can be described as ^40^:

$$\begin{aligned} Min \sum_{i} \sum_{k} C_{ik}Y_{ik}(\sum_{j} h_{ij})+\sum_{j} \sum_{m} C_{mj}Y_{jm}(\sum_{i} h_{ij})+ \alpha\sum_{i} \sum_{j} \begin{aligned} \\ {\sum_{k} \sum_{m} h_{ij}C_{km}Y}_{ik}Y_{jm} \# \end{aligned}\#\left( Eq2 \right) \end{aligned}$$

Subject to the following constraints:

1. $\sum_{k} X_{k}=p$, where $p$ is the exact number of selected hubs.
2. $\sum_{k} Y_{ik}=1 \forall i$, which means each non-hub is allocated to one hub.
3. $Y_{ik}\leq X_{k} \forall i,k$, which means that nodes are only located in the selected hubs.
4. $X_{k}=\left\{ \begin{aligned} 1 \\ & 0 \end{aligned} \right. \forall k, where 1 if k is a hub and 0 if not$.
5. $Y_{ik}=\left\{ \begin{aligned} 1 \\ & 0 \end{aligned} \right. \forall i,k, where 1 node i is allocated to the hub k 0 if not$.

However, this formulation has several limitations are:

- Assuming a fully connected graph,
- Prohibiting connection between two non-hub nodes, even if they are closer, forcing passing by a hub,
- Requiring the set of nodes to be the same as the set of hubs.

Willey and Salmon (2021) tackled these limitations and updated the formulation as follows ^3^:

$$\begin{aligned} Max \sum_{i} \sum_{j} \sum_{k} \sum_{m} Y_{ik}Y_{jm}h_{ij}^{km}\left( C_{ij}^{g}-\left( C_{ik}^{g}+C_{km}^{f}+C_{mj}^{g} \right) \right) \#\left( Eq3 \right) \end{aligned}$$

Where $C_{ij}^{g}$ is the cost of the flow $(i, j)$ on the nodes graph g, and $C_{km}^{f}$ is the cost of the flow on the hubs’ graph. Thereby to overcome previous limitations, the authors added the following constraints ^3^:

1. The $\alpha\ll1$, which reflects the assumption that the travel between hubs is much less important than the hub-node one. This constraint is vital because it shapes the optimization process to reflect the real-world priority of cost-effective node-to-hub connections, while allowing for flexibility in the network’s overall structure.
2. The $(k, m)$ are selected hubs for which $X_{k}=X_{m}=1$, the constraint *is* $C_{ik}Y_{ik}\leq C_{im}$ $\forall k,m$, and it means that users are more likely to use the hub if the nodes are close to their origins or destinations. This constraint ensures the model considers a crucial real-world factor: people generally prefer to use hubs that are closer to their starting point or their destination.
3. The assumption that each travel needs to pass through a hub is relaxed as the objective is to minimize the cost, hence trips between non-hub nodes are allowed when the cost is less than when a hub is involved. This constraint introduces flexibility into the model by allowing direct travel between non-hub nodes, which is crucial because it reflects the reality that sometimes the most cost-effective route doesn’t necessarily involve using a hub. Then $C_{ij}^{g}> C_{ik}^{g}+C_{km}^{f}+C_{mj}^{g}$
4. There is at least one node to function as the base, which means $\exists k\in\varphi, X_{k}=1$, where $\varphi$ is a set of large nodes. This constraint guarantees that there’s at least one node acting as a starting point within the network. This is important because, in any transportation system, there needs to be an origin for the flow of goods or travelers.

The model’s initial constraints (1-5) establish the foundation by requiring the selection of exactly ‘$p$’ hubs, ensuring each non-hub node is assigned to one hub, and restricting hubs to specific locations. The 6th constraint minimizes connection costs between nodes and hubs by setting a low ‘α’ value and allows for strategic hub-to-hub routing, acknowledging that the shortest route may not always be optimal. Constraints 7-9 enhance the model by prioritizing hubs near demand points, permitting direct connections between non-hub nodes for cost efficiency, and requiring at least one base node for routing flexibility. This study employs the uncapacitated single allocation $p$-median HLP model^3^ to strategically locate mobility hubs, aiming to support decarbonization goals through optimized hub placement.

Furthermore, the primary aim of the GA was to reduce travel time and enhance traffic flow efficiency by factoring in traffic flow dynamics. This study adeptly traces the progressive performance of the GA, monitoring the evolution of fitness values through each iteration, culminating a significant fitness value of 7.7e7, underscoring the effectiveness of the model. The model identified an optimal solution and four other near optimal solutions (Table 3), then the study selected the total districts appearing in the five solutions to be the final selected sites at this first stage of the mobility hub locations selection process.

Table 3: GA’s Five Best Solutions

|  | **Selected Districts (each per region)** |
| --- | --- |
| **Optimal** | (Myeong-dong; Yongsin-dong; Seogyo-dong; Yeouido; Yeoksam 1-dong |
| **Near Optimal** | Jongno 1, 2, 3, and 4 -ga-dong; Yongsin-dong; Seogyo-dong; Yeouido; Yeoksam 1-dong |
|  | Jongno 1, 2, 3, and 4 -ga-dong; Yongsin-dong; Sinchon-dong; Yeouido; Yeoksam 1-dong |
|  | Hangangno-dong; Yongsin-dong; Sinchon-dong; Yeouido; Samseong 1-dong |
|  | Hoehyeon-dong; Yongsin-dong; Seogyo-dong; Yeouido; Yeoksam 1-dong |

The selected sites consistently favor areas with high travel demand and density, a pattern highlighted in the results presented in Figure 1. For example, hubs in Seoul’s central region, known for their commercial and tourist prominence, are situated in zones dense with corporate establishments and vibrant shopping locales, signifying elevated travel requisites.


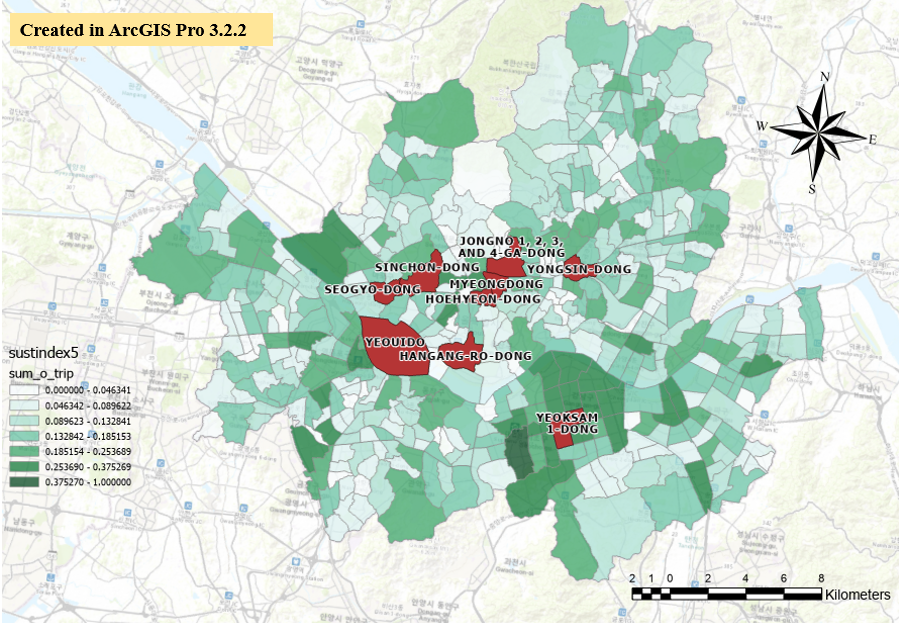


Figure 1: Genetic Algorithm Selected Districts

However, while the GA’s primary emphasis is on reducing travel time, it is essential to incorporate other facets of the built environment, like land-use diversity and transport infrastructure coverage. Therefore, the subsequent pivotal phase in pinpointing mobility hub locations is to undertake a suitability analysis, integrating other key variables in a manner that reflects their influences on carbon emissions. To achieve this, the study employs weights derived from the Ensemble methods, which are renowned for their robustness and exceptional predictive capability.

## Ensemble Methods

The ensemble method has gained prominence in diverse research fields due to its innate ability to amalgamate the strengths of various techniques, leading to enhanced robustness and accuracy ^41^. When applied to problems with multiple influencing factors, the ensemble approach stands out, as it is capable of handling high-dimensional and multicollinear data, identifying important factors, and providing accurate predictions ^42^.

Ensemble methods stand out in machine learning for their ability to significantly boost prediction accuracy beyond what’s possible with individual models ^43^. By harnessing the collective power of multiple models, these techniques can provide a more nuanced and comprehensive understanding of the data at hand. The reasons the ensemble methods are so effective are:

- Diversity and Comprehensive Insights: Individual models might inadvertently focus on specific aspects, potentially missing broader patterns. Ensemble methods, by combining diverse models, each with its unique lens on the data, ensure a more holistic analysis. This collective approach helps in capturing a broad spectrum of data characteristics, enhancing the predictive performance on unseen data ^44^.
- Combatting Overfitting: Overfitting is a common challenge in machine learning, where models perform well on training data but fail to generalize to new data. Ensemble methods address this by averaging out the predictions from various models. This process naturally reduces the noise and individual errors that single models may introduce, leading to a more robust model that performs better on unseen data ^44^.
- Bias and Variance Trade-off: Bias refers to errors from overly simplistic models, while variance refers to errors from models too sensitive to training data. Ensemble methods, especially those that aggregate high-variance models (like decision trees), can significantly lower variance without substantially increasing bias. This balance is crucial for building models that are both accurate and generalizable ^45^.
- Model Flexibility: One of the strengths of ensemble methods is their ability to integrate models of different types. This eclectic approach allows the ensemble to leverage the distinct strengths of each model type, potentially capturing both linear and nonlinear relationships within the data ^46^.
- Key Ensemble Techniques:
  - Bagging: This technique reduces variance by training multiple instances of a model on various subsets of the training data, then averaging their predictions. It’s particularly effective with high-variance models, such as decision trees, exemplified by the Random Forest algorithm ^47^.
  - Boosting: Boosting sequentially trains models, with each new model focusing on correcting the errors of its predecessors. The combined predictions of these models, weighted by their accuracy, often lead to a significant boost in performance. AdaBoost and Gradient Boosting Machines are prime examples, known for their ability to incrementally improve prediction accuracy ^45^.
  - Stacking: This technique involves training a meta-model to optimally combine the predictions of several base models. It leverages the diversity among the base models to achieve higher accuracy, illustrating the power of model integration ^45^.

The ensemble methods offer a powerful strategy for improving machine learning predictions through model diversification, error reduction, and the effective balance of bias and variance. Their ability to integrate multiple perspectives and methodologies makes them invaluable for tackling complex predictive tasks, though their applications should always consider existing computational resources and the specific nuances of the task at hand.

Before building the model, the dataset was split into two subsets: a training set and a testing set. The split was stratified to ensure an unbiased prediction, by preserving the proportions of the various classes in the target variable in both subsets. Specifically, 80% of the data was used for training and 20% was reserved for testing.

The ensemble methods employed in this study are renowned tools for supervised learning, each seeking to create predictive models rooted in training data. The Decision Tree Regressor, known for its simplicity, often grapples with overfitting. In contrast, the Random Forest approach amalgamates multiple trees, enhancing accuracy albeit at a heightened computational cost. Both AdaBoost and Gradient Boosting, while introducing added complexity, are tailored to bolster accuracy ^43^. Bagging, though adept at curtailing variance, might not always reach the accuracy pinnacle achieved by Random Forest ^48^. XGBoost, recognized for its prowess, demands considerable computational resources ^49^. Meanwhile, the KNeighbors Regressor, grounded in instance-based learning, gauges the proximity between instances ^50^. The MLP Regressor, a neural network-based approach, leverages interconnected layers of nodes to capture intricate patterns in data ^51^. Each method, in its essence, brings forth distinct strengths and challenges, collectively enriching the modeling landscape.

## Weights Calculation Method

To assess the significance of each feature in the dataset, this study computed their importance scores for every model in use. If the importance score is absent, the study resorted to calculating the permutation importance. Permutation importance is a methodology that transcends model specifics, evaluating how the performance of the model is impacted by shuffling the values of a particular feature ^52^. This permutation importance is calculated using the following formula (Eq4):

$$\begin{aligned} importance_{permutation}=performance_{noShuffling}-performance_{Shuffling} \#Eq4 \end{aligned}$$

Where:

- $importance_{permutation}$: is the permutation performance score.
- $performance_{noShuffling}$: the model’s performance on the original data.
- $performance_{Shuffling}$: the model’s performance on a dataset where the features’ values have been shuffled randomly.

Given the use of multiple models, we consolidated the feature importance scores across the models with the best performance to create a standardized mean importance score using the permutation method ^53^. This consolidation approach (Eq5) ensures that the resulting scores offer a comprehensive perspective, incorporating insights from each model, and are easily comparable across different models and datasets. Standardizing (Eq6) these importance’s scores involves placing them on a consistent scale, facilitating straightforward comparisons. This standardization process entails subtracting the mean importance score from each score and then dividing it by the standard deviation of all importance scores.

The consolidated mean importance score, which is a mean of importance scores for a factor $f$ across all models, can be represented by the following formula (Eq5):

$$\begin{aligned} \mu_{IS_{f}}= \frac{1}{n} \sum_{i=1}^{n} IS_{f,m_{i}} \#Eq5 \end{aligned}$$

Where:

- $\mu_{IS_{f}}$: the consolidated mean importance score
- $M=\{m_{1},m_{2},\ldots m_{n}\}$ : the selected ensemble methods
- $f$: a particulate factor
- $IS_{f,m_{i}}$ : the importance score of the factor $f$, using the model $m_{i}$

On the other hand, the standardization was conducted using the z-score normalization method, according to the following formula (Eq6):

$$\begin{aligned} SIS_{f,m_{i}}= \frac{IS_{f,m_{i}}- \mu_{IS_{f}}}{\sigma_{IS_{f}}} \#Eq6 \end{aligned}$$

Where:

- $SIS_{f,m_{i}}$: the standardized importance score for factor $f$ across all models $M$
- $IS_{f,m_{i}}$ : the importance score of the factor $f$ using the model $m_{i}$
- $\mu_{IS_{f}}$: the consolidated mean importance score for factor $f$ across all models $M$
- $\sigma_{IS_{f}}$: the standard deviation of the importance scores for factor $f$ across all models $M$

## Suitability Analysis

The previously calculated weight values play a crucial role in this phase of the second-stage selection process, which involves conducting suitability assessments to pinpoint the most sustainable hub locations. Using these weights, the study can assess and rank potential locations based on their suitability and contribution to the identified objectives. This step is vital for making informed decisions and optimizing the impact of the selected sites.

According to the results shown in Table 4, the five locations with the highest suitability scores are (1) Yeoksam 1-dong, (2) Myeongdong, (3) Yeouido, (4) Jongno 1, 2, 3, and 4-ga-dong, and (5) Hoehyeon-dong. However, it is recommended to strategically designate a mobility hub in each region, to ensure a comprehensive approach to mitigating car dependency and traffic congestion. These regional selections are rooted in their impressive performance within the suitability analysis and the diversity of characteristics they offer. Since the top five sites contain 3 within the central region alone, suitability scores will be compared by region. Hence, the final five selected sites, shown in Table 4 are Yeoksam 1-dong (east-south), Myeongdong (center), Yeouido (west-south), Seogyodong (west-north), and Yongsindong (east-north).

Table 4: Suitability Analysis Scores and Ranking

| Site (District) | Rank | Final Suitability Analysis score | Region |
| --- | --- | --- | --- |
| Yeoksam 1-dong | 1 | 0.59 | East_South |
| Myeongdong | 2 | 0.53 | Center |
| Yeouido | 3 | 0.5 | West_South |
| Jongno 1, 2, 3, and 4-ga-dong | 4 | 0.45 | Center |
| Hoehyeon-dong | 5 | 0.43 | Center |
| Seogyo-dong | 6 | 0.42 | West_North |
| Yongsin-dong | 7 | 0.4 | East_North |
| Hangang-ro-dong | 8 | 0.36 | Center |
| Sinchon-dong | 9 | 0.21 | West_North |

# References

1. Ratti, N. & El-Geneidy, A. Not all hubs are created equal: An analysis of future mobility hubs in the Greater Toronto Area. (2017).

2. Fan, B., Yang, Y. & Li, L. Integrated optimization of urban agglomeration passenger transport hub location and network design. *EURASIP J. Wirel. Commun. Netw.* **2018**, 168 (2018).

3. Willey, L. C. & Salmon, J. L. A method for urban air mobility network design using hub location and subgraph isomorphism. *Transp. Res. Part C Emerg. Technol.* **125**, 102997 (2021).

4. Arnold, T., Frost, M., Timmis, A., Dale, S. & Ison, S. Mobility Hubs: Review and Future Research Direction. *Transp. Res. Rec. J. Transp. Res. Board* 036119812211089 (2022) doi:10.1177/03611981221108977.

5. Michel, G., Rodas, D. & Büttner, B. Factors of the Built and Social Environments Associated with the Allocation of Mobility hubs: A Systematic Literature Review Chair of Urban Structure and Transport Planning Factors of the Built and Social Environments Associated with the Allocation of Mob. (2022).

6. Navratilova, K. *et al.* Application of Mobility Hub for automatic parking in the city. in *2021 Smart City Symposium Prague (SCSP)* 1–7 (IEEE, 2021). doi:10.1109/SCSP52043.2021.9447395.

7. Kirschner, F. & Lanzendorf, M. Parking management for promoting sustainable transport in urban neighbourhoods. A review of existing policies and challenges from a German perspective. *Transp. Rev.* **40**, 54–75 (2020).

8. Storme, T., Casier, C., Azadi, H. & Witlox, F. Impact Assessments of New Mobility Services: A Critical Review. *Sustainability* **13**, 3074 (2021).

9. Pase, F., Chiariotti, F., Zanella, A. & Zorzi, M. Bike Sharing and Urban Mobility in a Post-Pandemic World. *IEEE Access* **8**, 187291–187306 (2020).

10. Santos, G. Sustainability and Shared Mobility Models. *Sustainability* **10**, 3194 (2018).

11. Wang, G., Han, Q. & de vries, B. The multi-objective spatial optimization of urban land use based on low-carbon city planning. *Ecol. Indic.* **125**, 107540 (2021).

12. Lyu, G., Bertolini, L. & Pfeffer, K. How does transit-oriented development contribute to station area accessibility? A study in Beijing. *Int. J. Sustain. Transp.* **14**, 533–543 (2020).

13. Hachette, M. & L’Hostis, A. Mobility Hubs, an Innovative Concept for Sustainable Urban Mobility? in *Smart Cities.* 245–278 (Springer, Cham, 2024). doi:10.1007/978-3-031-35664-3_14.

14. Alumur, S. A. *et al.* Perspectives on modeling hub location problems. *Eur. J. Oper. Res.* **291**, 1–17 (2021).

15. Contreras, I. & O’Kelly, M. Hub Location Problems. in *Location Science* 327–363 (Springer International Publishing, 2019). doi:10.1007/978-3-030-32177-2_12.

16. O’kelly, M. E. The location of interacting hub facilities. *Transp. Sci.* **20**, 92–106 (1986).

17. Campbell, J. F. Integer programming formulations of discrete hub location problems. *Eur. J. Oper. Res.* **72**, 387–405 (1994).

18. Kovalchuk, M. L., Ushtan, P. I., Ushenko, Y. O. & Soltys, I. V. Advantages and Disadvantages of Learning a Multilayer Neural Network Using a Genetic Algorithm. *Optoelectron. Information-Power Technol.* **43**, 19–23 (2022).

19. So, J. (Jason) *et al.* Integrated Mobility Hub Location Selection for Sustainable Urban Mobility. *Sustain. Cities Soc.* **99**, 104950 (2023).

20. Pamucar, D. S., Tarle, S. P. & Parezanovic, T. New hybrid multi-criteria decision-making DEMATEL-MAIRCA model: sustainable selection of a location for the development of multimodal logistics centre. *Econ. Res. Istraživanja* **31**, 1641–1665 (2018).

21. Novotná, M., Švadlenka, L., Jovčić, S. & Simić, V. Micro-hub location selection for sustainable last-mile delivery. *PLoS One* **17**, e0270926 (2022).

22. Shahparvari, S., Nasirian, A., Mohammadi, A., Noori, S. & Chhetri, P. A GIS-LP integrated approach for the logistics hub location problem. *Comput. Ind. Eng.* **146**, 106488 (2020).

23. Peker, M., Kara, B. Y., Campbell, J. F. & Alumur, S. A. Spatial Analysis of Single Allocation Hub Location Problems. *Networks Spat. Econ.* **16**, 1075–1101 (2016).

24. Deluka-Tibljaš, A., Karleuša, B., Dragičević, N. & Aleksandra Deluka-Tibljaš, Ü. Review of multicriteria-analysis methods application in decision making about transport infrastructure Übersicht der Anwendung von Methoden der Multikriterien-Analyse bei der Entscheidungsfindung für die Verkehrsinfrastruktur. **65**, 619–631 (2013).

25. Anderluh, A., Hemmelmayr, V. C. & Rüdiger, D. Analytic hierarchy process for city hub location selection - The Viennese case. *Transp. Res. Procedia* **46**, 77–84 (2020).

26. Shin, H., Lee, T. & Lee, H.-R. Skyport location problem for urban air mobility system. *Comput. Oper. Res.* **138**, 105611 (2022).

27. Melnik-Leroy, G. A. & Dzemyda, G. How to Influence the Results of MCDM?—Evidence of the Impact of Cognitive Biases. *Mathematics* **9**, 121 (2021).

28. Alumur, S. & Kara, B. Y. Network hub location problems: The state of the art. *Eur. J. Oper. Res.* **190**, 1–21 (2008).

29. Campbell, J. F. *Theory and Methodology Integer programming formulations of discrete hub location problems*. *European Journal of Operational Research* vol. 72 (1994).

30. Gelareh, S. & Nickel, S. Hub location problems in transportation networks. *Transp. Res. Part E Logist. Transp. Rev.* **47**, 1092–1111 (2011).

31. Ishfaq, R. & Sox, C. R. Hub location-allocation in intermodal logistic networks. *Eur. J. Oper. Res.* **210**, 213–230 (2011).

32. Fan, T., Chiang, W.-C. & Russell, R. Modeling urban hazmat transportation with road closure consideration. *Transp. Res. Part D Transp. Environ.* **35**, 104–115 (2015).

33. Akbari, V., Sadati, M. E. H. & Kian, R. A decomposition-based heuristic for a multicrew coordinated road restoration problem. *Transp. Res. Part D Transp. Environ.* **95**, 102854 (2021).

34. Hosseini, M., MirHassani, S. A. & Hooshmand, F. Deviation-flow refueling location problem with capacitated facilities: Model and algorithm. *Transp. Res. Part D Transp. Environ.* **54**, 269–281 (2017).

35. Zeng, W., Miwa, T. & Morikawa, T. Application of the support vector machine and heuristic k-shortest path algorithm to determine the most eco-friendly path with a travel time constraint. *Transp. Res. Part D Transp. Environ.* **57**, 458–473 (2017).

36. Hsu, Y.-T., Yan, S. & Huang, P. The depot and charging facility location problem for electrifying urban bus services. *Transp. Res. Part D Transp. Environ.* **100**, 103053 (2021).

37. Greig, R. & Arranz, J. Generating Agent Based Models From Scratch With Genetic Programming. in *The 2021 Conference on Artificial Life* 64 (MIT Press, 2021). doi:10.1162/isal_a_00383.

38. An, L. *et al.* Challenges, tasks, and opportunities in modeling agent-based complex systems. *Ecol. Modell.* **457**, 109685 (2021).

39. Jang, J., Ju, X., Ryu, U. & Om, H. Coevolutionary Characteristics of Knowledge Diffusion and Knowledge Network Structures: A GA-ABM Model. *J. Artif. Soc. Soc. Simul.* **22**, (2019).

40. Hsieh, S.-Y. & Kao, S.-S. A Survey of Hub Location Problems. *J. Interconnect. Networks* **19**, 1940005 (2019).

41. Wang, L. *et al.* Improving the robustness of beach water quality modeling using an ensemble machine learning approach. *Sci. Total Environ.* **765**, 142760 (2021).

42. McCoy, D., Mgbara, W., Horvitz, N., Getz, W. M. & Hubbard, A. Ensemble machine learning of factors influencing COVID-19 across US counties. *Sci. Rep.* **11**, 11777 (2021).

43. Rozhkov, A. *et al.* Effectiveness variation of different census outreach activities: An empirical analysis from the state of Illinois using machine learning and user interface technologies for participatory data collection. *Appl. Geogr.* **154**, 102928 (2023).

44. Hao, T., Elith, J., Lahoz‐Monfort, J. J. & Guillera‐Arroita, G. Testing whether ensemble modelling is advantageous for maximising predictive performance of species distribution models. *Ecography (Cop.).* **43**, 549–558 (2020).

45. Chatzimparmpas, A., Martins, R. M., Kucher, K. & Kerren, A. StackGenVis: Alignment of Data, Algorithms, and Models for Stacking Ensemble Learning Using Performance Metrics. *IEEE Trans. Vis. Comput. Graph.* **27**, 1547–1557 (2021).

46. Wang, N., Zhou, W. & Li, H. Learning Diverse Models for End-to-End Ensemble Tracking. *IEEE Trans. Image Process.* **30**, 2220–2231 (2021).

47. Li, X. *et al.* Beds: Bagging Ensemble Deep Segmentation For Nucleus Segmentation With Testing Stage Stain Augmentation. in *2021 IEEE 18th International Symposium on Biomedical Imaging (ISBI)* 659–662 (IEEE, 2021). doi:10.1109/ISBI48211.2021.9433869.

48. Altmann, A., Toloşi, L., Sander, O. & Lengauer, T. Permutation importance: a corrected feature importance measure. *Bioinformatics* **26**, 1340–1347 (2010).

49. Gong, J., Chu, S., Mehta, R. K. & McGaughey, A. J. H. XGBoost model for electrocaloric temperature change prediction in ceramics. *npj Comput. Mater.* **8**, 140 (2022).

50. Nonaka, K., Shekkizhar, S. & Ortega, A. Graph-based Deep Learning Analysis and Instance Selection. in *2020 IEEE 22nd International Workshop on Multimedia Signal Processing (MMSP)* 1–6 (IEEE, 2020). doi:10.1109/MMSP48831.2020.9287121.

51. Zhu, H. Multi-layered perceptron and its applications in biotechnology. *Theor. Nat. Sci.* **20**, 165–171 (2023).

52. Winkler, A. M., Ridgway, G. R., Webster, M. A., Smith, S. M. & Nichols, T. E. Permutation inference for the general linear model. *Neuroimage* **92**, 381–397 (2014).

53. Li, K., Huang, G. & Baetz, B. Development of a Wilks feature importance method with improved variable rankings for supporting hydrological inference and modelling. *Hydrol. Earth Syst. Sci.* **25**, 4947–4966 (2021).
